# Retraction: Analysis and early warning management of resource and environmental carrying capacity in agricultural provinces: A case study of Henan Province

**DOI:** 10.1371/journal.pone.0324284

**Published:** 2025-05-09

**Authors:** The PLOS One Editors

The *PLOS One* Editors retract this article [1] due to concerns about peer review integrity, authorship, and potential manipulation of the publication process. These concerns call into question the validity of the reported results. We regret that the issues were not identified prior to the article’s publication.

ML did not agree with the decision. CW either did not respond directly or could not be reached.
